# Cardiac MR fingerprinting for T1 and T2 mapping in four heartbeats

**DOI:** 10.1186/1532-429X-18-S1-W1

**Published:** 2016-01-27

**Authors:** Jesse I Hamilton, Yun Jiang, Dan Ma, Bhairav B Mehta, Wei-Ching Lo, Yong Chen, Mark A Griswold, Nicole Seiberlich

**Affiliations:** 1 grid.67105.350000000121643847Biomedical Engineering, Case Western Reserve University, Cleveland, OH USA; 2 grid.241104.20000000404524020Radiology, University Hospitals, Cleveland, OH USA

## Background

MR Fingerprinting (MRF) (Ma et al, Nature, 2013) can potentially achieve shorter scan times than conventional mapping by exploiting flexible sequence parameters. Cardiac MRF has been reported for single-slice mapping of T1 and T2 in sixteen heartbeats (Hamilton et al, Proc ISMRM, 2015). Here the scan duration is reduced to four heartbeats using an iterative multi-scale denoising pattern recognition (Pierre et al, Magn Reson Med, 2015).

## Methods

The MRF scan is acquired during a breathhold and uses ECG triggering. Every heartbeat contains magnetization preparation and data readout stages. A non-selective inversion (TI = 21 ms) sensitizes the signal to T1 before the first excitation, and an MLEV composite T2-preparation is applied during the third (TE = 40 ms) and fourth (TE = 80 ms) heartbeats. The acquisition window employs a FISP readout with variable flip angles (4-15 deg), and the TR is minimized and held constant (5.1 ms) to maximize the number of images acquired each heartbeat. Data are sampled along a variable density spiral (TE = 1.4 ms) with 48 interleaves and a golden angle interleaf ordering. Each heartbeat, 48 images are collected in a 245 ms scan window and gridded using the NUFFT with acceleration factor R = 48. A dictionary of signal timecourses was calculated using a Bloch simulation, and quantitative maps were made using the denoising algorithm with 4 iterations (Gaussian weights of kmax*0.05, kmax*0.25, kmax*0.4, and no filtering on the final iteration).

Eleven volunteers were imaged in short-axis orientation on a 3T Siemens Skyra with 30 coils. Scans were performed using MRF, the original 17-heartbeat MOLLI, and a 10-heartbeat T2-prepared bSSFP sequence (TE = 0, 25, and 55 ms). All scans used a 192 × 192 matrix, 1.6 × 1.6 × 8.0 mm^3^ spatial resolution, and 300 mm2 FoV. The conventional maps were fit by a nonlinear parameter estimation. Mean and standard deviations in T1 and T2 were computed over the entire myocardium and assessed using a Bland-Altman analysis. Additionally, in one volunteer the MRF scan was concatenated to map 4 slices during a 16-heartbeat breathhold, with each slice acquired in 4 consecutive heartbeats.

## Results

**Figure 1:**
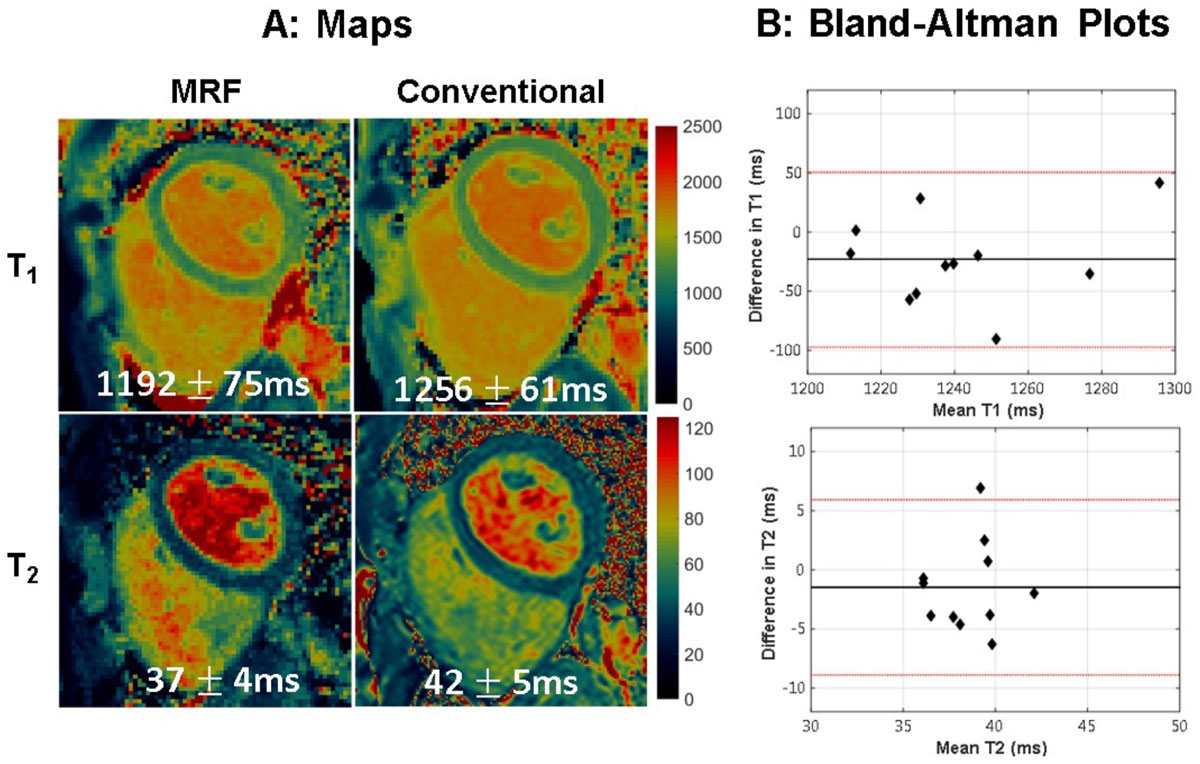
**1A: Maps of T1 and T2 acquired with 4-heartbeat MRF (left) compared with MOLLI and T2-prepared bSSFP (right)**. The mean and standard deviations for T1 and T2 within the myocardial wall are also presented. 1B: Bland-Altman plots comparing myocardial T1 between MRF and MOLLI and T2 between MRF and T2-prepared bSSFP in 11 volunteers. The 95% limits of agreement are indicated by red lines.

**Figure 2:**
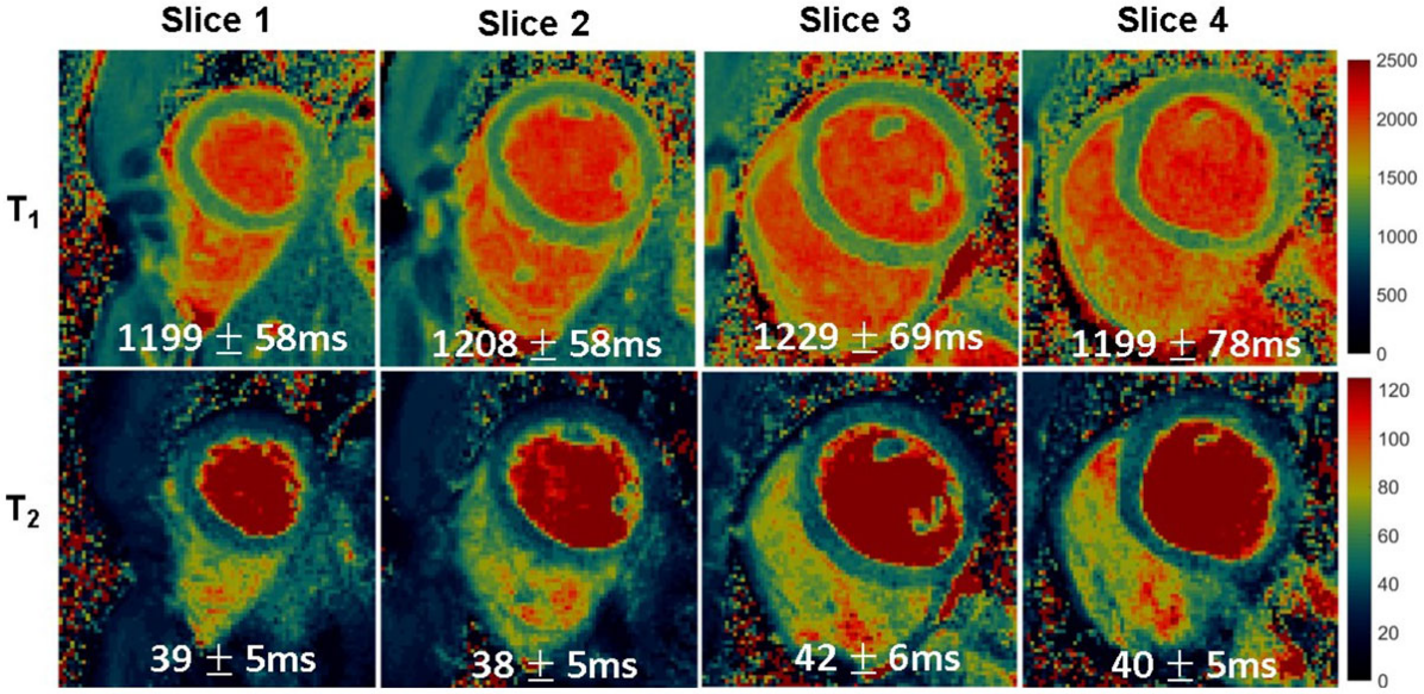
**MRF T1 and T2 maps from a volunteer acquired from four slices during a breathhold lasting sixteen heartbeats, where each slice was acquired in four consecutive heartbeats**. The mean and standard deviations for T1 and T2 within the myocardial wall are also given.

Representative maps are in shown in Figure 1A, where the myocardial T1 and T2 from MRF are in good agreement with conventional maps. From the Bland-Altman plots (Figure 1B), the T1 measurements for all volunteers lie within the 95% limits of agreements (-97 ms, 51 ms) with bias -23 ms. Ten out of eleven T2 measurements are within the 95% limits of agreement (-8.9 ms, 5.9 ms) with bias -1.5 ms. Figure 2 displays MRF maps from 4 slices collected during a breathhold lasting 16 heartbeats (4 heartbeats per slice).

## Conclusions

Myocardial T1 and T2 maps can be acquired in 4 heartbeats using MRF with iterative denoising pattern recognition. The short scan time could be useful for patients who have difficulty with breathholds and could enable increased coverage if combined with multi-slice techniques.

